# AI-IoT driven system for agricultural pest outbreak risk prediction

**DOI:** 10.1038/s41598-026-54478-0

**Published:** 2026-05-26

**Authors:** Jean Pierre Nyakuri, Celestin Nkundineza, Omar Gatera, Kizito Nkurikiyeyezu, Gervais Mwitende

**Affiliations:** 1 African Centre of Excellence in Internet of Things (ACEIoT), College of Science and Technology, University of Rwanda, Kigali, Rwanda; 2 Department of ICT, Rwanda Polytechnics-Gishari College, Rwamagana, Rwanda; 3 Department of Mechanical and Energy Engineering, University of Rwanda, Kigali, Rwanda; 4 Department of Electrical and Electronics Engineering, Rwanda Polytechnics-Gishari College, Rwamagana, Rwanda

**Keywords:** Explainable Artificial Intelligence (XAI), Convolutional Neural Network (CNN), Fuzzy logic, Edge device, Precision agriculture, Pest stage detection, Energy science and technology, Engineering

## Abstract

Invasive pests pose a significant threat to agricultural production, particularly maize crops, with severe implications for food security. Timely detection of pest development stages and accurate prediction of outbreak risks are essential for effective management. This study introduces a hybrid model combining Explainable Artificial Intelligence (XAI), a lightweight Convolutional Neural Network (CNN), and Fuzzy Logic (FL) for Fall Armyworm (FAW) detection and weather-based risk prediction. The model uses Tiny-MobileNet-SE for image classification, Grad-CAM for interpretability, and FL inference based on environmental parameters. Tiny-MobileNet-SE achieved 98.6% accuracy, 98.5% F1-score, 98.6% recall, a compact size of 0.72 MB, and 80 ms latency on Raspberry Pi 5, outperforming state-of-the-art lightweight models including EfficientNetB0, SqueezeNet, MobileNet-v2, MobileNet-v3, and ShuffleNet. The proposed system delivers a power-efficient, scalable, and user-friendly solution for precision agriculture, providing actionable insights for pest management and supporting sustainable crop protection strategies.

## Introduction

 The agriculture sector plays a crucial role in global economic stability and food security, as over 45% of the global population and 70% in Africa rely on agriculture for both sustenance and economic stability^1^. However, the threats posed by invasive agricultural pests, particularly the Fall Armyworm (Spodoptera frugiperda, FAW), has become a significant global challenge. The FAW, a destructive pest known for its rapid reproduction and ability to devastate crops like maize, rice, wheat, sugar cane and sorghum, has spread to over 44 countries in Africa, since its initial outbreak from the Americas^2^. To minimize the damage caused by FAW and other invasive pests, early detection and timely intervention are critical.

**Table 1:** FAW pest development stages and their activities.

Stage	Image	Duration	Favorable climate condition	Behavior and effect crop
Egg		2–3 days	Warm temperatures and moderate humidity (20–30 °C, 50–70% RH)	**Inactive** with minimal effect on crop
Larva		14–22 days	Warm and humid conditions (20–30 °C, 70–80% RH)	**Very active** with severe damage on crop
Pupa		8–30 days	Warm and moist soil conditions (20–30 °C, 40–60% RH)	**Inactive** and minimal effect on crop
Adult or moth		10–21 days	Warm conditions dry or moderate humidity (20–28 °C, 30–60% RH),	**Active** in flying, and laying eggs. Indirect effects on crop

Currently, pest management is still relying heavily on physical inspection and chemical pesticides application^3^. While these methods can be effective, they are often labor-intensive, time-consuming, difficult for large farms, and may cause harmful environmental impacts^4^. These manual inspection methods are often prone to human errors and results in delayed detection, especially when dealing with fast-spreading pests like the FAW, allowing the infestation to reach critical levels before appropriate actions are taken. Another common approach is a widespread use of chemical pesticides before invasive pests can invade agricultural fields^5^. However, this approach is related to overuse of toxic chemicals, which are expensive for smallholder farmers and leads to soil degradation, water pollution, and harm to non-target organisms including beneficial insects like bees. Therefore, more efficient and sustainable pest management solutions based on advanced technologies are urgently needed^6^.

The promising alternative to traditional methods is the integration of Artificial Intelligence (AI), Machine Learning (ML), and Internet of Things (IoT) technologies in precision agriculture^7,8^. AI models, particularly Convolutional Neural Networks (CNNs), have shown remarkable success in automating pest identification by analyzing real-time images and recognizing patterns that indicate the presence of pests^9,10^. Additionally, Internet of things (IoT) technology-based devices equipped with cameras, sensors, and connectivity modules enable real-time field data collection and processing, allowing remote control and informed decision making. Furthermore, IoT networks allow for continuous data collection from the field, providing real-time updates on environmental conditions such as temperature, humidity, and rainfall, which are critical for predicting pest behavior and outbreaks^11^. By integrating AI and IoT, agricultural pest management has been transformed by shifting from reactive to proactive strategies, allowing for early detection, targeted interventions, and reduced reliance on chemical pesticides^12,13^.

Environmental factors such as weather patterns and soil condition influence the proliferation of pests, leading to variations in pest development rates, persistence, reproduction, and outbreak dynamics^14^. AI and IoT technologies are used to predict pest outbreaks. For instance, ML models can analyze temperature, humidity, and rainfall patterns to forecast the possibility of pest emergence and determine potential risk periods for crop infestation^15^. Leveraging environmental conditions and images of plants offers High accurate prediction of pest outbreaks^16^. CNN models are effective in analyzing plant images to detect the presence of pests and their development stages (as described in Table 1), while Fuzzy Logic (FL) techniques can interpret imprecise environmental data to assess outbreak risks, mimicking human reasoning in uncertain scenarios. However, AI models like CNN operate as black box and lack transparency, making them difficult to trust on decision they make. To address this challenge, Explainable AI (XAI) techniques such as Grad-CAM are employed to visualize the internal decision-making process of CNNs, highlighting the image regions that most influenced the model’s output. Integration of image processing and environmental prediction model increases accuracy in predicting pest outbreaks.

In this study, we present an innovative hybrid AI model that combines the strengths of a lightweight CNN (Tiny-MobileNet-SE) model, Fuzzy Logic inference, and XAI methods to detect and predict pest outbreak, specifically Fall Armyworm (FAW). The CNN model detects and classifies FAW’s development stages using images captured from the field. Grad-CAM-based XAI technique is incorporated to highlight image regions that contributed to the model’s classification, enhancing interpretability and transparency. Furthermore, The FL inference module simultaneously evaluates weather parameters, including temperature, humidity, and rainfall to assess the risk of FAW outbreak. The resulting hybrid model is deployed on a Raspberry Pi 5, where it demonstrates real-time performance with 98.6% classification accuracy and low latency of 80 ms, while preserving a small memory footprint of 0.72 MB, making it suitable for edge deployment.

The main contributions of this work include: (i) the development and optimization of a lightweight CNN “Tinty-MobileNet-SE” model for detection and classification of FAW development stages; (ii) the integration of fuzzy inference for FAW outbreak risk assessment based on environment parameters; and (iii) the integration of explainability features using Grad-CAM to provide the model transparency. This combination of CNN, fuzzy reasoning, and explainability techniques provides a robust, accurate, power-efficient, and interpretable tool for real-time pest monitoring. Furthermore, the deployment of this compact hybrid model in Raspberry pi 5 makes it a practical toll suitable for edge deployment.

## Related works

Various researches in the fields of AI and ML domain have been conducted to strive technological solutions for addressing challenges of invasive pests^17^. AI-powered model using the Inception-ResNet-V2 framework was proposed by S. Vyas et al.^18^, achieving 99.54% accuracy in identifying soybean pests. F. Ali et al.^19^ and Q. Dong et al.^20^ used object detection CNN models for precisely detect agricultural pests, realizing 82.43% and 90.7% mAP, respectively. Similarly, Vision Transformer (ViT) algorithms were used for various crop pest classification. Enhanced Vision Transformer (EViTA) was proposed in^21^, and compared with CNNs model for different Peanut crop pests using IP102 Dataset. Furthermore, F. Maniraguha in^22^ and S. Farian in^23^ used CNN algorithms to detect FAW pests affecting Maize crops. While these AI-based models performed well for pest images classification, they are computationally intensive, making the less suitable for resource-constrained deployment.

The IoT has revolutionized precision agriculture, particularly pest management by enabling the continuous collection of real-time data from the field, enabling informed decision making and remote control with less human effort^24^. C. J. Chen et al.^25^, G. Garg et al.^26^ and S. Yang et al.^27^ integrated IoT with AI based on CNN algorithms for identifying and control various pests and diseases affecting crops, specifically Maize crop. Furthermore, IoT sensors combined with AI algorithms can alert farmers when certain environmental conditions, such as temperature and humidity are conducive to pest outbreaks^28^. However IoT-based systems are facing various challenges related to limited communication fractures in remote and rural areas for real-time data transmission, making cloud computing difficult to adopt^29^. Therefore, these limitations highlight the need for edge computing solutions that can process data locally, without relying on cloud services^30^.

IoT-edge computing devices assisted by Unmanned Aerial Vehicles (UAVs) offer a promising solution to the challenges of pest detection in agriculture^31^. C.-J. Chen et al.^32^ integrated a Tiny-YOLOv3 model with an UAV embedded system NVIDIA Jetson TX2 for real-time identification of T. papillosa in the orchard field. Similarly, F. Ramoliya et al.^33^ proposed Drone assisted TL-AGRO AI system, integrating Edge IoT device and CNN architectures including VGG16, XceptionV2, ResNet50V2 for. These systems detect and classify insects in agricultural fields, providing timely alerts and pesticide recommendations to enhance productivity and sustainability in precision agriculture.

Explainable AI (XAI) approaches such as Grad-CAM have been applied to visualize and interpret the interior decision-making process of CNNs in pest detection tasks, allowing users to recognize which image regions influence predictions^34^. Bragadeeshwaran et al. in^35^ used VGG19 and XAI to classify 18 different classes of pests in jute, achieving 92% accuracy. Similarly, Grad-CAM based XAI integrated with CNN models were applied to detect and describe pest in^10^. Furthermore, Fuzzy Logic (FL) has proven effective in analyzing environmental uncertainty, including vague or imprecise inputs such as temperature, humidity, and rainfall to predict the likelihood of pest outbreaks^36^. Various studies have used fuzzy C-means to classify pest affecting crops^37,38^.

While various approaches have been explored for pest detection and control in agriculture, including traditional image recognition and object detection techniques based on DL/CNN, IoT, and UAV-assisted systems, number of challenges such as accuracy, explainability, and scalability in resource-constrained environments^39^. In our study, we developed a hybrid AI model that combines the strengths of a lightweight CNN (Tiny-MobileNet-SE) model, Fuzzy Logic inference, and XAI technique to detect and predict pest outbreak, specifically Fall Armyworm (FAW). The CNN model detects and classifies FAW’s development stages using images captured from the field. Grad-CAM-based XAI technique is incorporated to highlight image regions that contributed to the model’s classification, enhancing interpretability and transparency. We also incorporate a fuzzy inference system to assess the potential rest of pest outbreak based on weather conditions.

## Methodology

This section details the design process used to develop the proposed hybrid AI model that combines lightweight Convolutional Neural Networks (CNN), Explainable AI (XAI), and Fuzzy Logic (FL) for real-time pest detection and outbreak risk prediction. The methodology is divided into the following phases: dataset preparation, image-based pest detection using Tiny-MobileNet-SE, integration of explainability using Grad-CAM, fuzzy logic-based outbreak prediction based on environmental parameters, and deployment on an edge device (Raspberry Pi 5).

### System architecture of the AI-powered pest monitoring

**Fig. 1:**
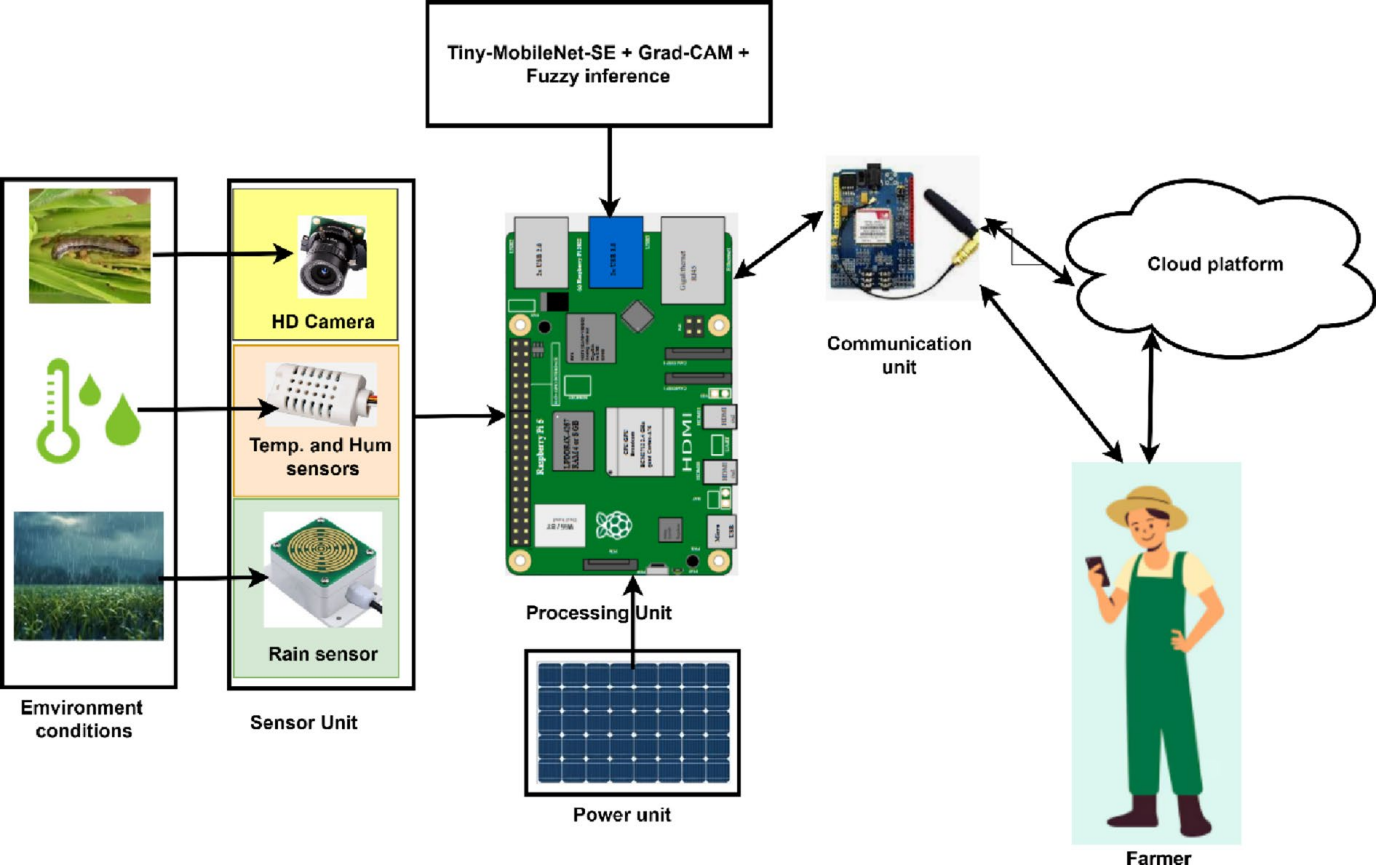
System architecture of the AI-powered pest monitoring.

The overall system architecture as illustrated in Fig. 1, is designed for on-site pest detection and outbreak risk prediction. It is comprised of three main functional units: The sensing unit for environmental parameters collection and pest image acquisition, the processing unit for pest development stage classification and weather conditions forecasting, and communication unit for remote data transmission and farmer notification.

A. **Sensing layer**.

The sensing layer consists of multiple sensors, including HD camera, temperature, relative humidity, and rain sensors deployed in the field to capture both environmental conditions and crop pest status:

- *HD Camera sensor*: it is used high-resolution crop image acquisition from the field.
- *Temperature and Humidity Sensors*: they collect ambient temperature and relative humidity data, which are critical parameters that influence pest proliferation and spread.
- *Rain and snow Sensor*: it measures rainfall conditions favorable for pest development to provide relative weather data to support pest outbreak predictions.

B. **Processing Unit**.

**Table 2:** Sensor components specifications.

Sensors	Technical Specifications
HD Camera	5MP resolution, 2592 × 1944 pixels, 1080p video at 30 FPS, f2.4 lens, 1.85 mm focal length, 70° (standard) FOV, and 72.60 × 24 mm dimensions.
Temperature and Humidity (DHT 22)	3 to 5 V power, 2.5 mA max current, 0–100% humidity readings with 2–5% accuracy, −40 to 80 °C temperature readings ± 0.5 °C accuracy, 0.5 Hz sampling rate, 27 mm x 59 mm x 13.5 mm body size, and 3 wires 23 cm long.
Rain Sensor and snow sensor	10 ~ 30 V DC power, 7.5 W(heating), heating start-up temperature: <15 °C, maximum heating temperature: 40 °C (default), Operating temperature: −40 °C ~ 60 °C.

At the core of the system is a Raspberry Pi 5, acting as the main edge computing device. It processes incoming environmental and crop pest data from the sensors and camera using:

- Tiny-MobileNet-SE: A lightweight CNN model designed for real-time pest multi-classification on resource-constrained edge devices.
- Explainability AI based on Grad-CAM (Gradient-weighted Class Activation Mapping) that Generates visual explanations for the CNN’s decision-making to enhance interpretability for field users.
- Fuzzy Inference System (FIS): For assessing the pest outbreak level, FIS is integrated. It employs environmental sensor data, specifically temperature, humidity, and rainfall to estimate the likelihood of a pest outbreak.

C. **Communication Layer**.

At this layer data is exchanged between the system and users (farmers) via GSM/GPRS communication module. Farmers can get insights of their farm conditions through both cloud-based applications and cellular network-based notification.

### Dataset description

To train and evaluate the CNN model, a dedicated image dataset was constructed from Plantvillage repository images^40^ and images captured from the fields. The data was labeled according to FAW pest development stages, namely: egg, larva, pupa, and adult moth. These stages were selected according to their agronomic importance and damaging impact on crops. The Table 2, and Table 3 describes the dataset pre-processing.

**Table 3:** Dataset description.

Dataset	No. of images	No. of classes	Source
FAW pest dataset	24,800	4	PlantVillage, field image collection

**Table 4:** Image pre-processing, dataset augmentation, and training details.

Processing technique	Details
Image resizing	224 × 224 pixels
Width and Height Shifts	Up to 0–30%
Shear	Up to 30%
Zoom	Up to 30%
Brightness Adjustments	[0.8, 1.2]
Horizontal Flips	Applied randomly
Fill Mode	Nearest
Dataset splitting	70% training, 15% validation, 15% testing
Optimizer	Adam
Learning rate	3 × 10^− 4^
Atch size	64
Epochs	150 (with early stopping, patience = 20)
Hardware for training	Raspberry pi 5

### Lightweight CNN model “Tiny-MobileNet-SE” design

**Fig. 2:**
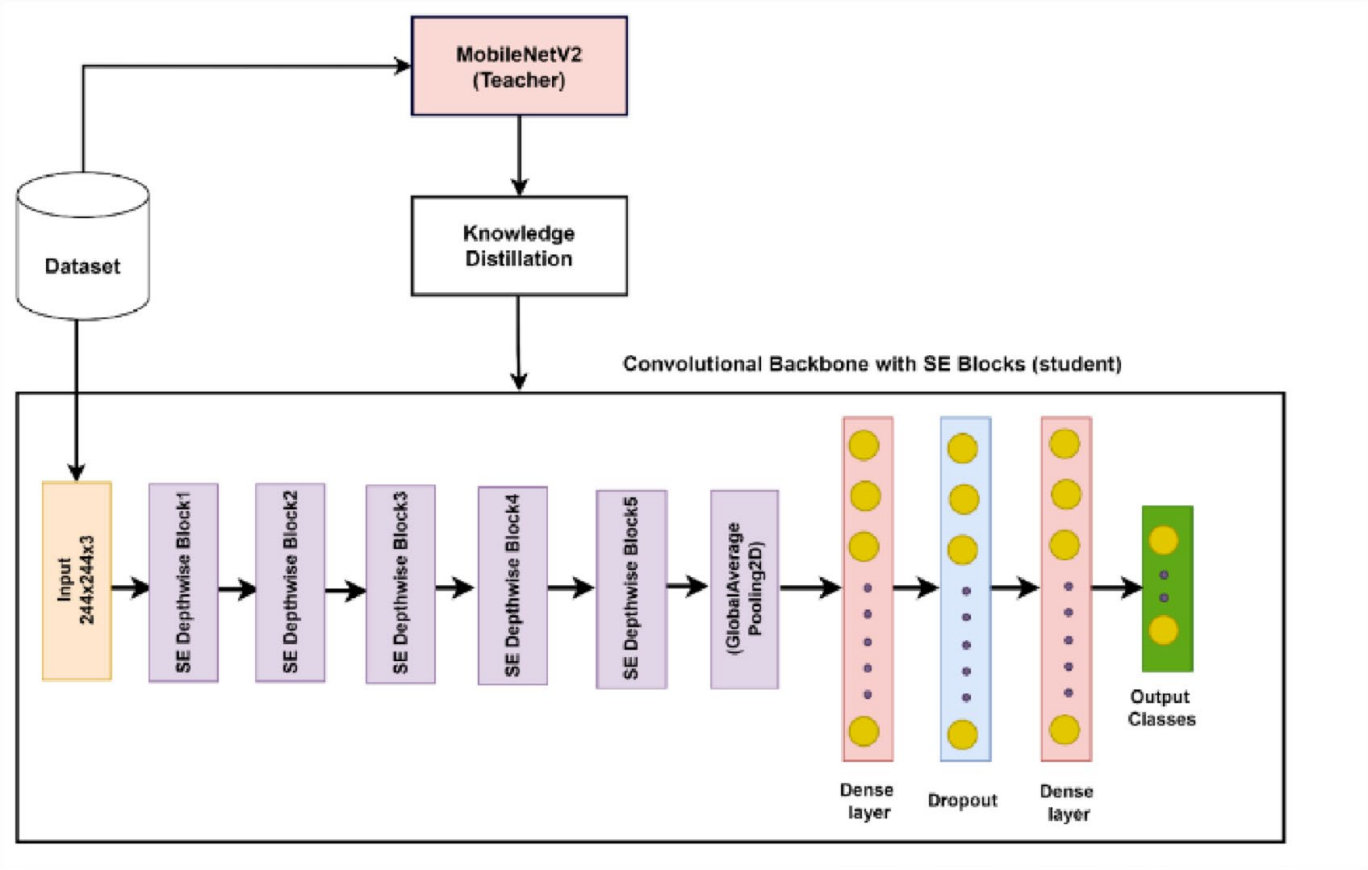
Tiny-MobileNet-SE architecture.

The Tiny-MobileNet-SE architecture is built on three main components, including pre-trained MobileNetv2 model (Teacher model), Convolutional Backbone with SE Blocks (student), and Knowledge Distillation process as depicted in Fig. 2.

The designed lightweight architecture “Tiny‑MobileNet‑SE” is designed to be an ultra‑lightweight student model delivered from MobileNet-v2. It preserves the core efficiency principles of depthwise‑separable convolutions (DSC) while integrating channel‑wise attention via Squeeze‑and‑Excitation (SE) modules. The structure adopts a efficient configuration consisting of five (5) consecutive SE‑enhanced depthwise blocks, each built from a 3 × 3 depthwise convolution followed by an SE recalibration module with a reduction ratio (between 8 and 16), and a subsequent 1 × 1 pointwise convolution for channel projection. To significantly reduce computational complexity, all convolutional layers are scaled using a depth multiplier α = 0.35, leading to considerably compressed intermediate structure relative to the MobileNetV2 as a teacher model. The block of five SE‑depthwise units is followed by a global average pooling layer and a lightweight classification head comprising a 128‑unit fully connected layer, dropout regularization, and a final softmax output. This configuration reduces significantly the number of parameters (1 M parameters reduction) while maintaining sufficient expressive capacity to benefit from soft‑label supervision during knowledge distillation.

### Explainable AI (XAI) with Grad-CAM

**Fig. 3:**
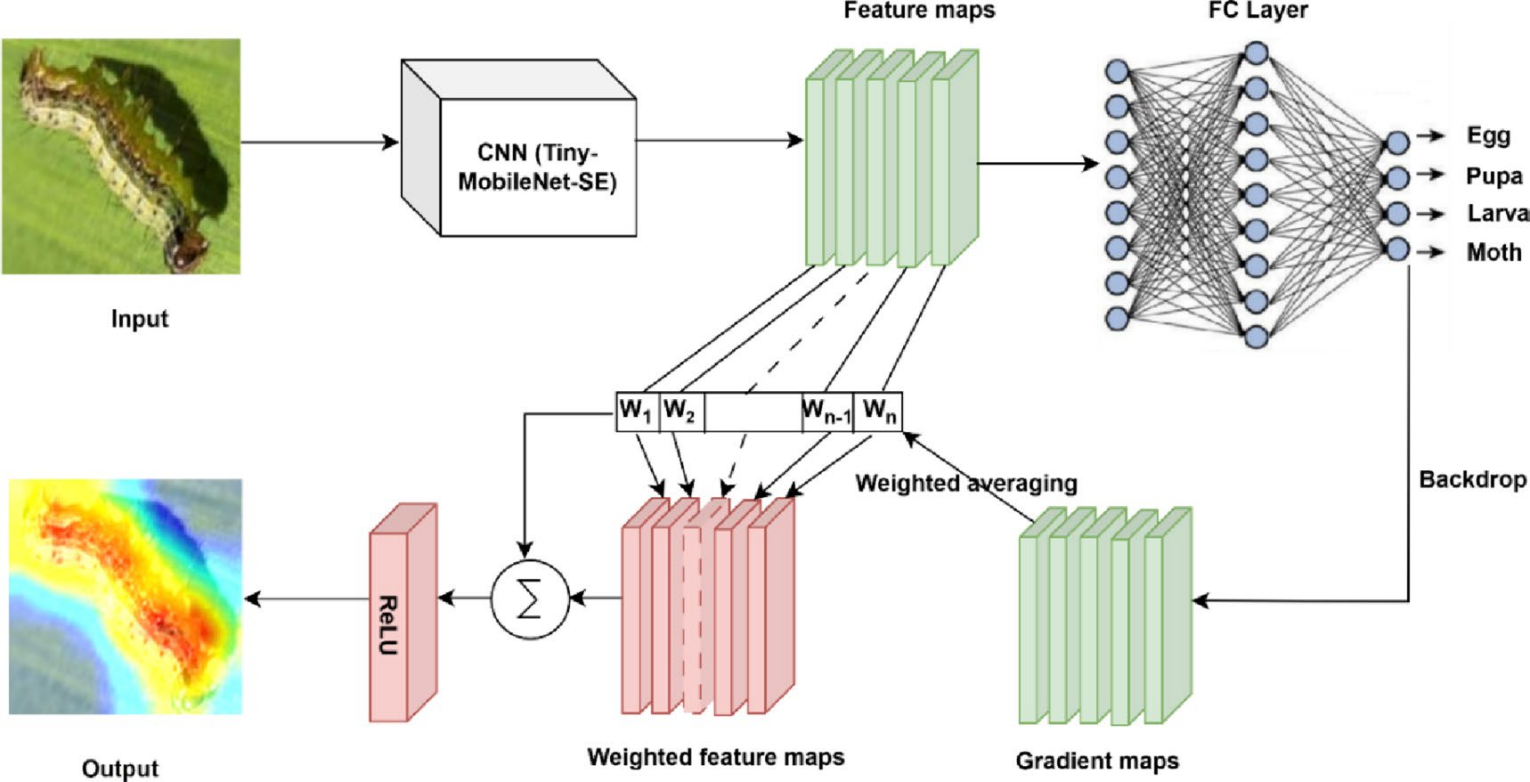
Grad-CAM visualization process for the Tiny-MobileNet-SE model.

To enhance the transparency of the CNN model results, the explainability based on Gradient-weighted Class Activation Mapping (Grad-CAM) as shown in Fig. 3 was added. The workflow illustrates how the model highlights the most important spatial features influences the decision-making process.

1. **Input Image and Feature Extraction**.

The process begins by capturing image of the plant affected by a pest. This image is passed through the CNN (Tiny-MobileNet-SE) model, which extracts convolutional features maps, specifically, the final convolutional layer outputs that preserve information (features) critical for class prediction.

Let the input RGB image be denoted as $\:I\in\:{R}^{H\mathrm{*}W}$, where H and W are the image height and width, respectively. This image is passed through the model to generate prediction logits for each class. The convolutional backbone of the CNN model extracts intermediate feature maps (A):

1 $$\:A=\left\{\begin{array}{c}{A}^{1}{,A}^{1},{\dots\:,A}^{k}\},{A}^{k}\in\:{R}^{u*\nu\:}\end{array}\right.\}$$

Where:

K = Number of channels.

u × v = feature map’s spatial resolution.

To get the score for Target Class, the output logits are passed to a softmax layer to obtain the probabilities. If there is class c, the pre-softmax score $\:{y}^{c}$ is calculated by:

2 $$\:{y}^{C}={f}^{C}\left(A\right)$$

2. **Prediction and Gradient Computation**.

The final feature maps are flattened and forwarded to the fully connected (FC) layer for final prediction. The class activation maps are generated by computing the gradient (G) of the target class score with respect to the feature maps of the last convolutional layer. These gradients determine how every spatial location within the feature maps impacts class prediction.

3 $$\:\frac{\partial\:{y}^{C}}{\partial\:{A}_{ij}^{k}}\forall\:{k}_{,i,j}$$

3. **Weighted Feature Aggregation**.

The importance weights for each feature map are obtained by averaging gradients and compute the weighted sum of these feature maps. This combination is linear, and it emphasizes the areas that strongly influences the predicted class. Then, a ReLU activation is applied to retain only the parts that positively influence the prediction. To get the importance weights ($\:{\alpha\:}_{k}^{c}$). the gradients are pooled spatially using global average pooling.

4 $$\:{\alpha\:}_{k}^{C}=\frac{1}{Z}\sum\:_{i=1}^{u}\sum\:_{j=1}^{v}\frac{\partial\:{y}^{u}}{\partial\:A\begin{array}{c}k\\\:ij\end{array}}$$

Where:

Z = u*v.

4. **Output Heatmap generation and 0verlay**.

The resulting class activation map is normalized and converted into a heatmap. As shown in the bottom-left of Fig. 2, the heatmap uses a color gradient (e.g., red to blue) to indicate attention intensity, where red highlights the most influential regions. The heatmap is then superimposed onto the original input image, offering an intuitive visual interpretation of the model’s focus areas during prediction. The weighted sum ($\:{L}_{Grad-CAM}^{C}$) of the feature maps provides the class-discriminative localization map:

5 $$\:{L}_{Grad-CAM}^{C}=ReLU\left({\sum\:}_{k=1}^{k}{\alpha\:}_{k}^{C}{A}^{k}\right)$$

The ReLU operation retains only features that positively influence the class of interest. To overlay the heatmap on the input image, the localization map is upsampled ($\:{\widehat{L}}^{c}$):

$$\:{\widehat{L}}^{c}=$$*Upsample* ($$\:{L}_{Grad-CAM}^{C},\:size=(H,W)$$) (6)

7 $$\:Heatmap(i,j)=\:\frac{{\widehat{L}}^{c}\left(i,j\right)-\mathrm{m}\mathrm{i}\mathrm{n}\left({\widehat{L}}^{c}\right)}{\mathrm{max}\left({\widehat{L}}^{c}\right)-\mathrm{m}\mathrm{i}\mathrm{n}\left({\widehat{L}}^{c}\right)}$$

### Fuzzy logic-based outbreak risk estimation

Pest outbreak is significantly influenced by environment conditions. Therefore, a Fuzzy Inference System (FIS) was integrated into the proposed system to estimate the risk level of pest outbreak based main environmental parameters, including temperature, humidity, and rainfall. The system models the uncertainty and nonlinear relationships between environmental factors and pest development stages determined by CNN model, enabling situation-based decision support for early intervention and providing recommendations.

#### Fuzzy system inputs and output

**Fig. 4:**
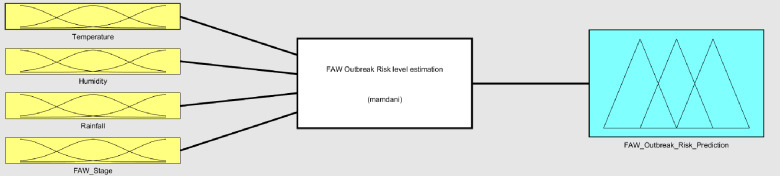
input and output of the proposed Fuzzy Logic Inference System for FAW Outbreak Risk Estimation.

The system has four input parameters, namely temperature, humidity, rainfall, and phase of the pest development, enabling more adaptive and intelligent decision-making in pest monitoring and control as described in Fig. 4; Table 4.

**Table 5:** Fuzzy Inputs System, input/output and their membership.

Temperature (^O^C)		Humidity (%)		Rainfall (mm)		Pest development stage		Outbreak Risk level	
Range	Linguistic variable	Range	Linguistic variable	Range	Linguistic variable	Range	Linguistic variable	Range	Linguistic variable
> 20	Low	> 30	Low	> 80	Low	0	Egg	> 35	Low
20–30	Medium	30–60	Medium	80–180	Medium	1	Pupa	35–65	Medium
30<	High	60<	High	180<	High	2	Larva	65<	High
	-		-		-	3	Moth	-	-

#### Fuzzy IF-THEN rule base

**Fig. 5:**
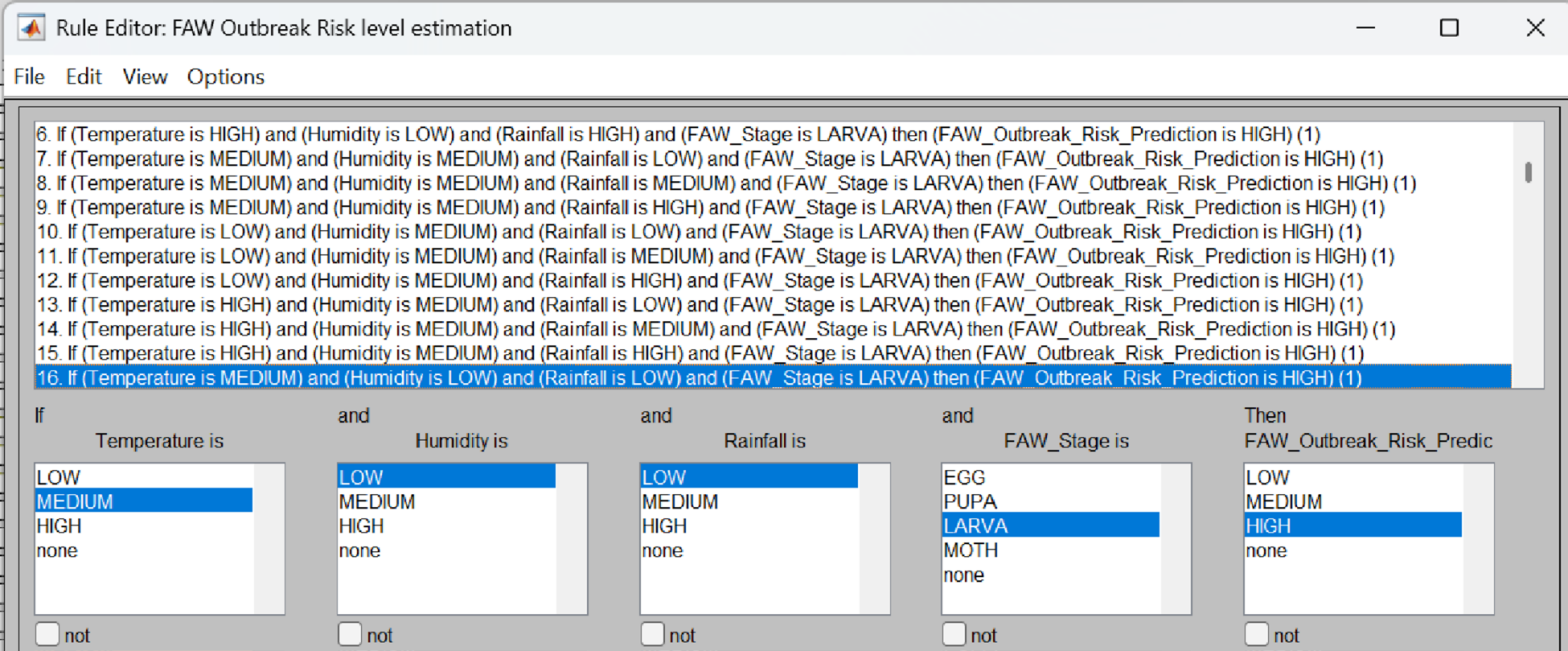
Fuzzy Rule development.

**Fig. 6:**
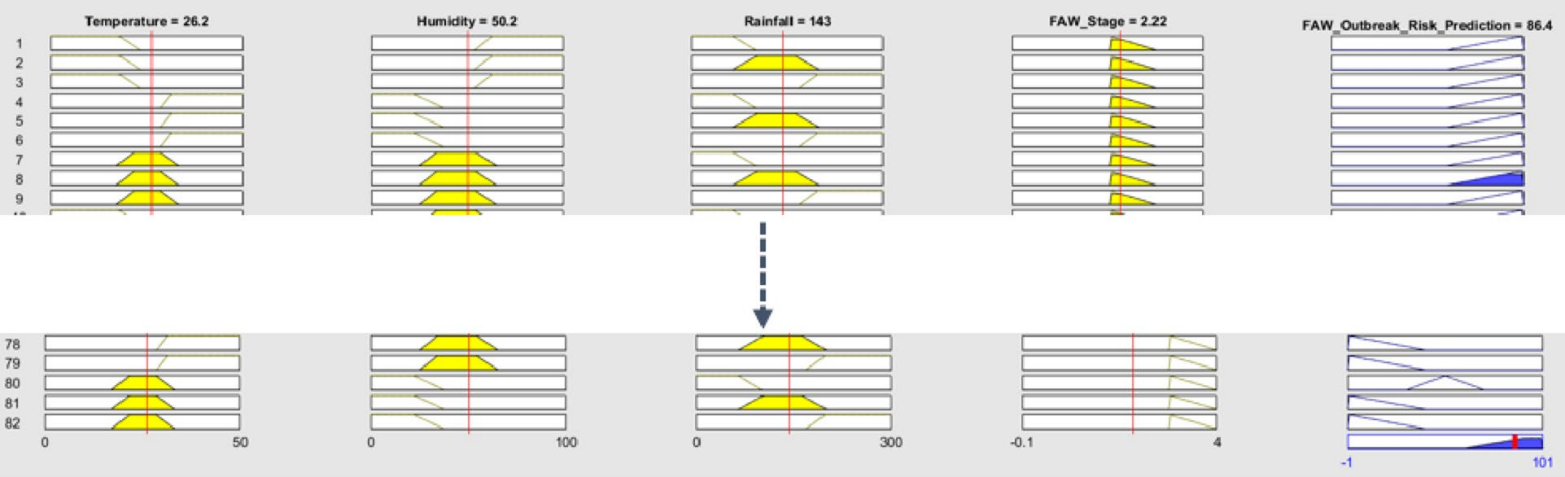
Fuzzy Rule view.

A set of IF-THEN rules was developed for the Fuzzy Inference System, basing on expert knowledge and heuristic rules to infer the risk level of a FAW outbreak. These rules integrate linguistic variables corresponding to environmental parameters such as temperature, humidity, rainfall, as well as the pest development stages. The rules were initially constructed and tested using MATLAB’s Fuzzy Logic Toolbox as illustrated in Figs. 5 and 6 to simulate system behavior and ensure logical consistency. After validation, this set of rules was implemented in Python (TensorFlow lite) for integration into the final decision support system on Raspberry pi 5.

#### Pseudocode for the system

**Fig. 7:**
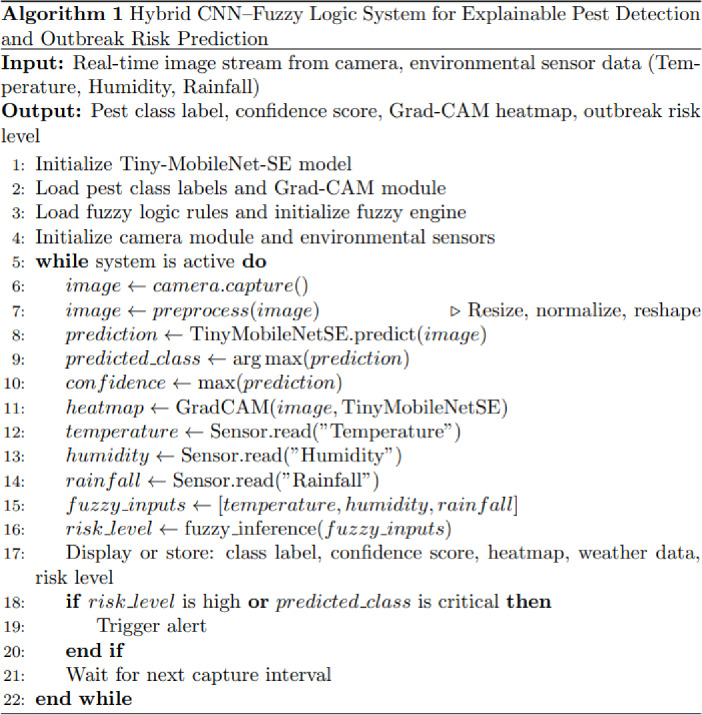
Pseudocode for the system.

As illustrated in Fig. 7, the pseudocode outlines the workflow of the proposed Hybrid CNN-Fuzzy-XAI framework for Pest Detection and Outbreak Risk Prediction. The algorithm begins with capturing real-time image stream from camera, which is then processed by the lightweight Tiny-MobileNet-SE model that classifies images in their respective classes. To ensure output transparency, the system integrates an Explainable AI (XAI) component using Grad-CAM, highlighting the key regions contributing to the model’s decision. Finally, a Fuzzy inference system is employed for potential pest outbreak risk level assessment based on environmental parameters. Based on the combined outcomes, the system provides interpretable recommendations to support informed agricultural decision-making.

## Results and discussion section

This section presents the performance evaluation of the proposed hybrid model in terms of pest stage classification accuracy, explainability AI using Grad-CAM, fuzzy logic-based outbreak risk prediction, benchmarking against state-of-the-art lightweight CNN models.

### Performance of the Tiny-MobileNet-SE Model for pest stage detection

The Tiny-MobileNet-SE model was trained and evaluated on a dataset of Fall Armyworm (FAW) pest development stages, including egg, larva, pupa, and adult moth. The model achieved an overall classification accuracy of 98.6%, with an F1-score of 98.5%, recall of 98.6%, and model size of 0.72 MB on the validation set. Addition The Tiny-MobileNet-SE model was compared with state of the state-of-the-art lightweight models trained on the same dataset as presented in Table 5.

**Table 6:** Comparison of Tiny-MobileNet-SE with traditional CNN architectures.

Model name	Accuracy	F1-score	Recall	Size (MB)	Parameter (million)	5-Fold Cross Validation Accuracy	Inference Time (ms)
Tiny-MobileNet-SE	98.6%	98.5%	98.6%	0.72	0.48	97.1%	80

The small difference between the single validation accuracy (98.6%) and the 5‑Fold Cross‑Validation accuracy (97%) or a 1.5% gap, reflects natural variation across different data partitions as well as subtle variations in image features among pest developmental stages.

**Fig. 8:**
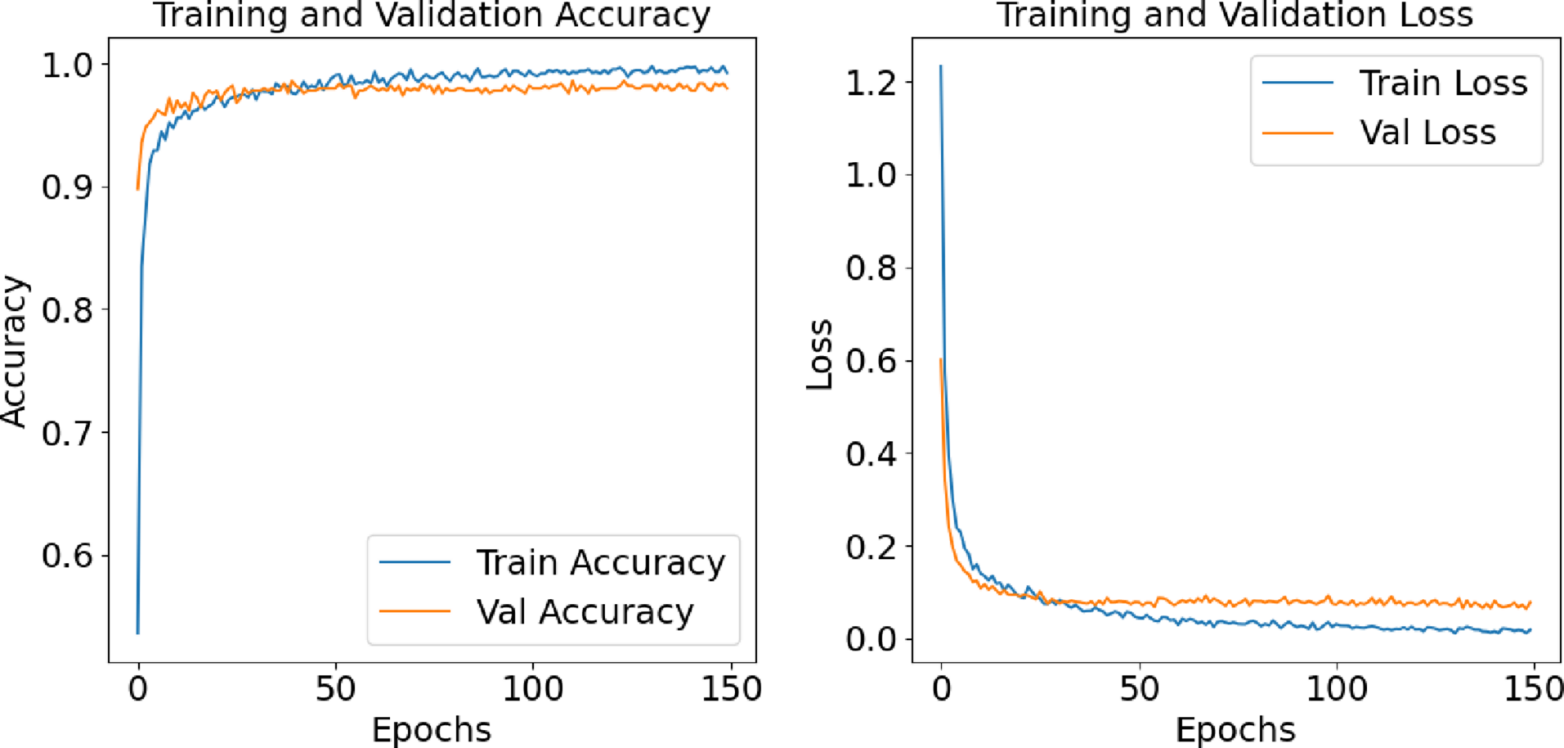
Training Vs validation accuracies and raining Vs validation losses curves.

**Fig. 9:**
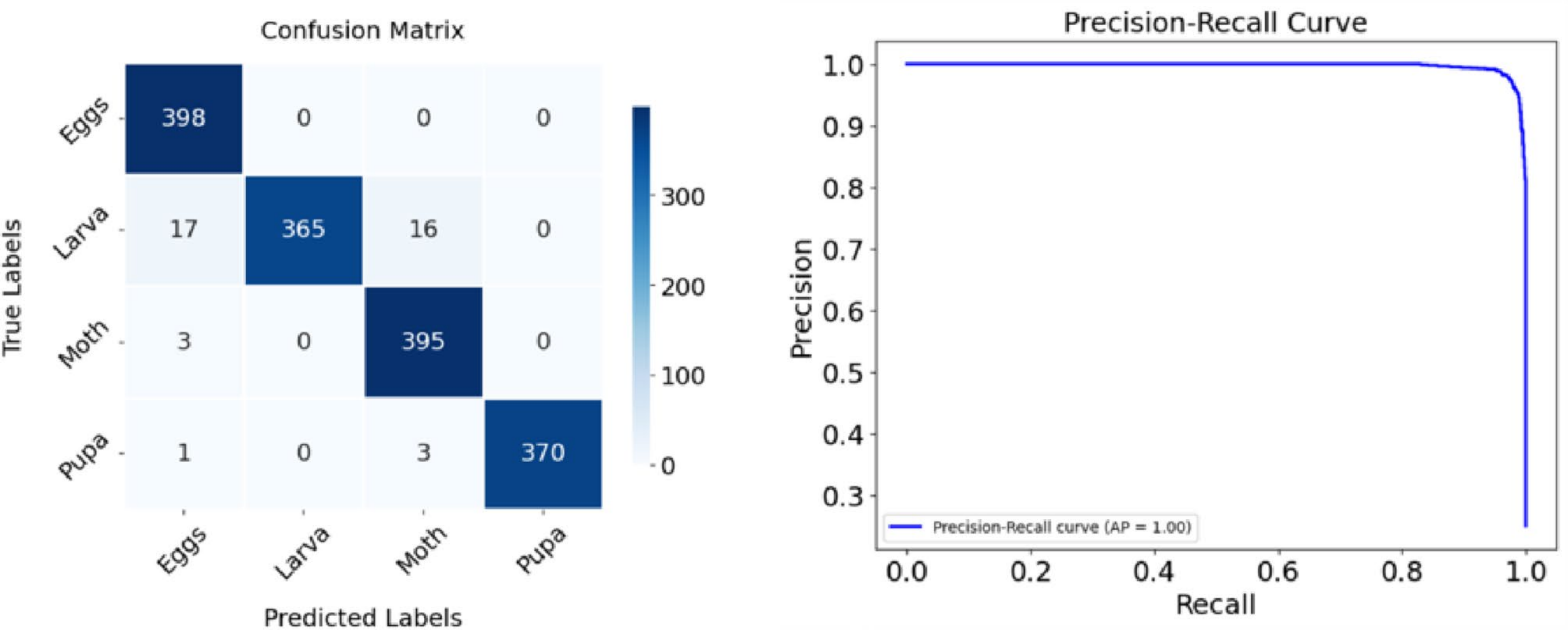
Confusion metrics, and precision-Recall curve, respectively.

As illustrated in Fig. 8, the model’s performance graphs show the variation of the Tiny-MobileNet-SE model’ accuracies and losses (training and validation) over 100 training epochs. While the Fig. 9 presents the confusion matrix for multi-class classification results with the corresponding precision-Recall curve, respectively.

While the Tiny-MobileNet-SE model achieved high classification accuracy overall, the confusion matrix reveals a biologically significant misclassification pattern such as 17 Larvae misclassified as eggs and 16 as moth among 365 tests samples. This misclassification is due to overlapping visual features in the dataset, impacting of the Larva stage on crop damage. To overcome this limitation, a targeted data augmentation, class-weighted loss functions, and extended field image collection with a robust DH camera to improve stage-specific accuracy.

### Explainability through Grad-CAM visualization

**Fig. 10:**
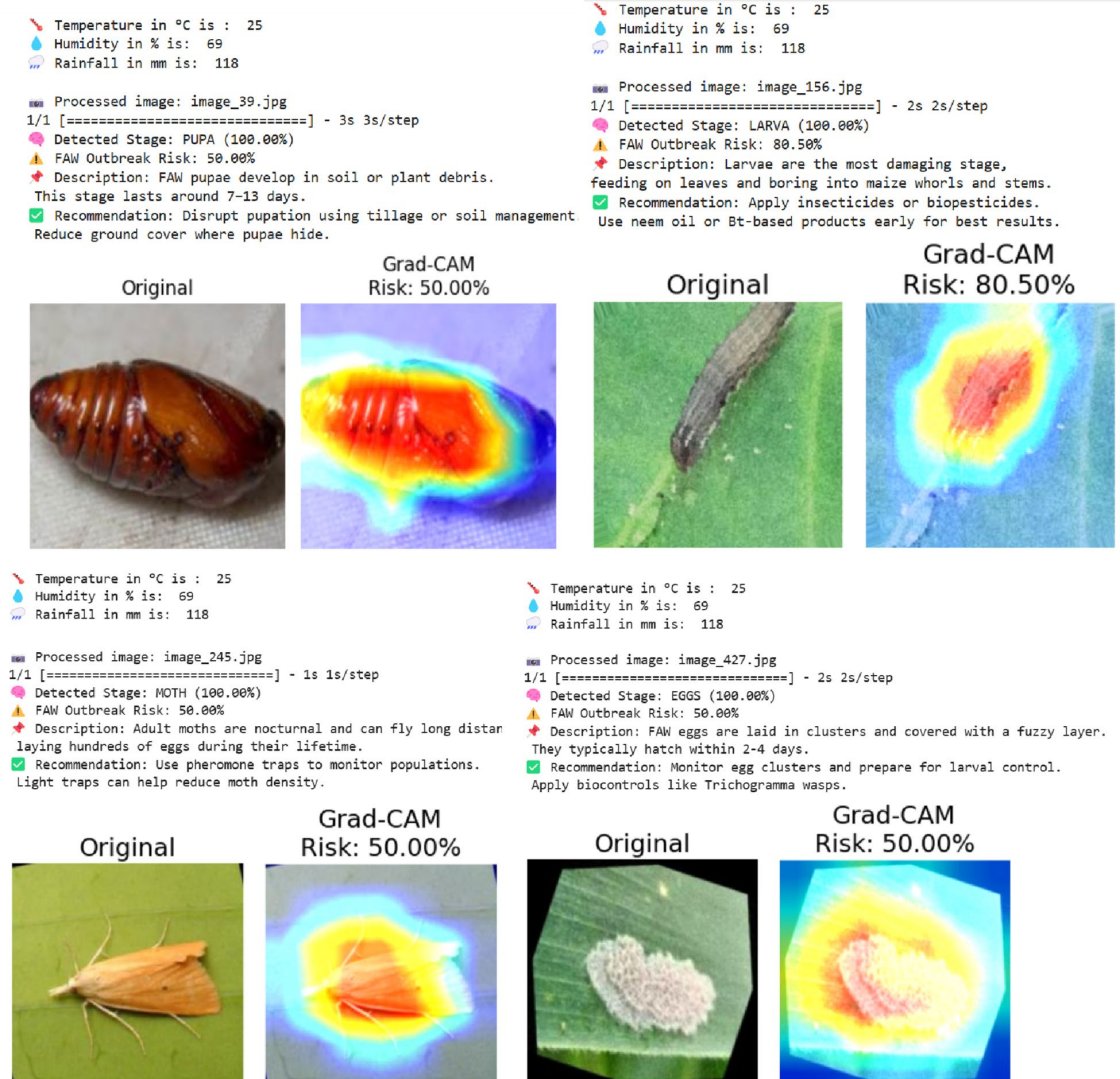
results of the model with original image (left), Grad-CAM image with highlighted region (right), environmental parameters, risk level prediction, description of the image, and recommendations.

A layer of Explainability was incorporated to enhance interpretability of the CNN model, indicating the most influencing area on an image. The Grad-CAM technique was integrated to visualize the areas of input images that the CNN model focused on during classification. Results presented in Fig. 10, show that the Grad-CAM heatmaps correctly highlighted biologically relevant regions associated with pest characteristics within image.

### Fuzzy logic inference for outbreak risk prediction

**Fig. 11:**
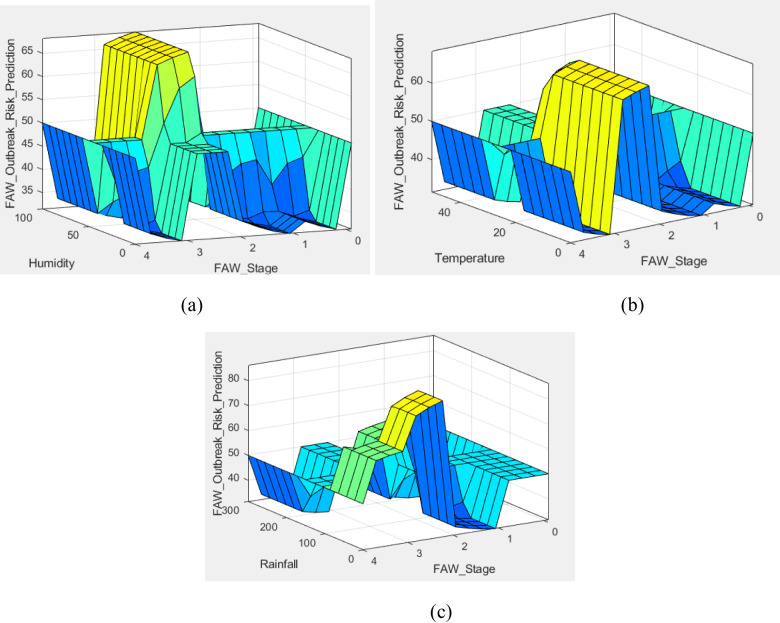
(**a**. **b**, and **c**) present the FAW outbreak risk level prediction with respect to humidity, temperature, and rainfall, respectively.

To assess the Risk level of the pest existence, a fuzzy logic (FL) module was evaluated using simulated and real environmental data, including temperature, humidity, and rainfall. The inference system classifies the FAW outbreak risk into four levels: Low, Moderate, and Severe. The Fig. 11a, b, and c illustrates FAW outbreak risk level prediction with respect to humidity, temperature, and rainfall, respectively.

### Discussion

In this study, a hybrid AI system that combines a lightweight CNN model, Explainable AI based on Grad-CAM, and a Fuzzy Inference System was proposed and validated to detect and assess the FAW pest outbreak risk level. The CNN model accurately classifies FAW pest development stages, including egg, pupa, larva, and adult moth. While the Fuzzy inference system assesses the pest outbreak levels from the weather conditions.

The developed Tiny-MobileNet-SE model achieved 98.6% classification accuracy, 98.5% F1-score, and 80 ms latency on Raspberry Pi 5 with a compact model size of only 0.72 MB. Compared to other state-of-the-art lightweight models such as EfficientNetB0, MobileNet_v2, ShuffleNet, and SqueezeNet, the Tiny-MobileNet-SE consistently outperformed them in terms of performance accuracy and response time as presented in the Table 6.

**Table 7:** Comparison of Tiny-MobileNet-SE with traditional CNN architectures.

Model name	Accuracy	F1-score	Recall	Size (MB)	Parameter (million)	5-Fold Cross Validation Accuracy	Inference Time (ms)
EfficientNetB0	97.1%	97.5%	97.5%	18	16.2	98.2%	217
Squeezenet	97%	97.2%	97.2%	2.7	1.2	98.4%	105
MobileNet_v3	98.8%	98.7%	98.8%	8.2	5.1	98.6%	121
MobileNet_v2	98.9%	98.9%	98.8%	7	3.5	90%	112
ShuffleNet	96.2%	96.1%	96.3%	2	0.86	89.8%	96
**Tiny-MobileNet-SE**	**98.6%**	**98.5%**	**98.6%**	**0.72**	**0.48**	91%	**80**

Alternative models such as MobileNet-v2 and MobileNet-v3 achieve slightly higher accuracy of 98.9% and 98.8%, respectively, compared to Tiny-MobileNet-SE (98.6%). However, these models exhibit significantly larger sizes (7 MB and 8.2 MB, respectively) and increased parameter counts (3.5 M and 5.1 M), resulting in longer response times (112 ms and 121 ms), making them less suitable for resource-constrained deployment (Table 7).

These results highlight the efficiency of the model’s architecture and its suitability for real-time deployment in resource-constrained environments, particularly in remote farming regions where power and connectivity are limited.

Furthermore, for transparency purpose, an Explainable AI (Grad-CAM) is integrated with CNN model to identify the parts of the image that mostly influence the decision-making of the CNN model. Finally, a Weather-Based Pest Outbreak Risk Assessment Using Fuzzy Logic is integrated to effectively predict the potential risk of pest outbreaks. The Fuzzy Logic bridges the gap between crisp numerical weather data (temperature, humidity, and rainfall) and qualitative risk categories (e.g., Low, Medium, High FAW outbreak risk). This component models expert human reasoning under uncertainty and enables the system to deliver actionable insights on possible pest outbreaks.

These results highlight the efficiency of the model’s architecture and its suitability for real-time deployment in resource-constrained environments, particularly in remote farming regions where power and connectivity are limited.

## Conclusion

This study presents an innovative hybrid AI model integrating a CNN “Tiny-MobileNet-SE” model, Fuzzy Inference System, and Explainable AI based on Grad-CAM for real-time detection of Fall Armyworm pest and weather-driven outbreak prediction. The proposed model achieved impressive classification accuracy of 98.6%, 98.5% F1-score, 98.6% Recall, 0.72 MB size, and 80 ms inference time when deployed on Raspberry pi 5. Additionally, an Explainable AI is added on the classified image for transparency using Grad-CAM technique. Furthermore, a Fuzzy Inference System is integrated to assess the level of pest outbreak based environmental parameters, including temperature, humidity, and rainfall. This hybrid Explainable AI-driven CNN–Fuzzy Logic model provides a scalable, power-efficient, and interpretable edge-based system for precision pest management suitable for deployment in remote and limited internet connectivity.

Despite significant achievements in this area, several challenges remain. First, several environmental factors such as soil conditions and wind speed for more accurate outbreak prediction are still underexploited. The future work will involve scaling the system to integrate additional environmental parameters such as soil conditions and wind speed, expanding deployment on mobile or UAV-based platforms, and integrating a lightweight Large Language Model (LLM) for generating like human text to enhance explainability. These extensions will strengthen the model’s scalability, adaptability, and real-world impact across diverse agricultural landscapes.

## Data Availability

The datasets used and/or analyzed during the current study are available from the corresponding author upon reasonable request.
